# The Ocular Signature of TBI: A Narrative Review of Structural Changes and Potential Candidate Biomarkers

**DOI:** 10.3390/life16091430

**Published:** 2026-08-28

**Authors:** Sultan Alotaibi

**Affiliations:** 1 Optometry Department, College of Applied Medical Science, King Saud University, P.O. Box 10219, Riyadh 11433, Saudi Arabia; salotabi1@ksu.edu.sa or s.alotaibi@unsw.edu.au; 2 School of Optometry and Vision Science, University of New South Wales, Kensington, Sydney, NSW 2033, Australia

**Keywords:** TBI, orbital fractures, photosensitivity, ocular surface, pupillary response

## Abstract

Background: Traumatic brain injury (TBI) damages brain tissues and affects the ocular system. Ocular manifestations such as orbital fractures, ocular surface disease, photosensitivity, impaired pupillary response, and retinal damage often co-occur with TBI. Despite established diagnostic criteria, a gap remains, particularly in detection, monitoring, and recovery prediction. Since ocular manifestations co-occur with TBI, they may offer non-invasive means to address this gap. Accordingly, this review evaluates whether TBI-associated ocular manifestations can serve as biomarkers for TBI detection, monitoring, and recovery. Methods: A literature search was conducted across three databases for studies that address ocular manifestations of TBI listed above. Results: Orbital fractures occur in 69%, matching TBI severity. Occult fractures may indicate unseen mild TBI on advanced neuroimaging. Dry eye disease prevalence is inconsistent (15 and 37%). Tear film proteomics show altered inflammatory and repair proteins, though this area remains understudied. Vitreous total tau and neurofilament light chain correlate with brain levels in chronic traumatic encephalopathy. Photosensitivity affects approximately 50% of acute TBI and declines to 14% after three months. NPi-200 pupillometer shows high specificity in triaging acute injury but with low sensitivity. PLR-3000 pupillometer and smartphone applications require further validation. Retinal imaging shows biphasic thickening then progressive thinning, modulated by sex, severity and duration. Conclusions: No single ocular biomarker fulfills all roles. Instead, different markers map into distinct stages of the clinical course. Future research should prioritize molecular markers in the tear film, combined with changes in corneal nerves across different TBI severities.

## 1. Introduction

Traumatic brain injury (TBI) is defined as a “bump, blow or jolt to the head, or penetrating head injury, that results in disruption of the normal function of the brain” [1,2,3]. It represents a significant cause of disability and death in young adults, affecting 60 million individuals globally with increasing prevalence [4]. Common causes include falls, sports-related injuries, traffic accidents, assaults, and explosions [1]. As neuronal damage extends to multiple body systems, patients may suffer from immediate and long-term complications, including physical, behavioral, perceptual and cognitive impairment, sleep disturbance, gut dysmotility, visual disorder, and disruption of normal development in affected children [5,6]. These complications may be intensified in individuals who experience repeated TBIs and may also develop chronic traumatic encephalopathy (CTE), a neurodegenerative disease which can be fatal [7]. TBI also has been linked to the development of other neurodegenerative diseases and is considered a potential trigger for Alzheimer’s disease and Parkinson’s disease [8,9,10].

Primary TBI occurs immediately following the traumatic event, damaging neurons, glial cells, and the associated vasculature. If a brief loss of consciousness accompanies it, it is considered a concussion, which is a mild form of TBI. The severity of the concussion symptoms can be assessed using the Sport Concussion Assessment Tool (SCAT5) [11], and the post-concussion symptom scale (PCSS) [12]. Both assess systemic and cognitive symptoms. Mild TBI is occasionally undiagnosed due to the invisibility of injury, lack of loss of consciousness, and absence of abnormal findings in neuroimaging. Unconsciousness lasting for more than several minutes may be caused by moderate or severe TBI and may be accompanied by cerebral contusion.

Secondary TBI develops after the primary injury within minutes to hours, and this depends on the injury severity, patient response, and the effectiveness of medical intervention [13,14,15]. Secondary TBI is triggered by a complex cascade of pathophysiological events, including neuronal cell damage and disruption of the blood–brain barrier (BBB), accompanied by excitotoxicity. Excitotoxicity is among the earlier events and results from pathological accumulation of extracellular glutamate, leading to depolarization of the cell membrane and the influx of sodium and calcium ions. Calcium accumulation impairs mitochondrial function and generates reactive oxygen species, thereby damaging the cytoskeleton, cell membrane, and DNA, which may ultimately lead to apoptosis. Necrotic cells release damage-associated molecular patterns (DAMPs), which activate pattern recognition in glial cells, triggering a neuroinflammatory response and release of inflammatory cytokines. Concurrent mitochondrial dysfunction and energy depletion further perpetuate the inflammatory response, leading to disruption of the BBB and subsequent neutrophil invasion and expansion of the lesion zone [16].

TBI is diagnosed based on established diagnostic criteria including mechanism of injury, clinical assessment using symptom-based and cognitive tools, and neurological deficits. Once diagnosed, TBI is classified according to the Glasgow Coma Scale (GCS) as mild (13–15), moderate (9–12), and severe (3–8) [17]. Other clinical manifestations, including loss of consciousness and post-traumatic amnesia, may support the diagnosis. Imaging technologies, including computed tomography (CT) and magnetic resonance imaging (MRI) play an essential role in the diagnosis and prediction of outcomes in those with TBI [18]. A non-contrast CT scan is the mainstay in acute settings for detecting intracranial hemorrhage, whereas MRI has greater sensitivity for detecting non-hemorrhagic contusions and shear-strain injuries. Furthermore, blood-based biomarkers have been recently approved by the FDA and can be used to detect brain damage, including glial fibrillary acidic protein (GFAP) and ubiquitin C-terminal hydrolase L (UCH-L1). This should be determined within the first 24 h of the injury [19,20].

GFAP is a cytoskeletal monomeric protein found in astrocytes in the central nervous system and other extracranial tissues. It supports astrocytes and maintains the integrity of the BBB and synapses. It is detectable in the blood within one hour of the head injury, with a half-life of 24–48 h, and remains elevated for several months afterward. GFAP has been proposed as a sensitive biomarker of BBB disruption, and its level correlates with GCS score and brain imaging findings in acute settings [21]. UCH-L1 is a deubiquitinating enzyme found in neurons that targets proteins for metabolism or damaged proteins resulting from oxidation. It is detectable in the blood within 1 h and peaks after 8 h, with a half-life of 7–9 h. Combining GFAP and UCH-L-1 has been shown to provide high sensitivity and specificity for diagnosing TBI. Furthermore, it has been reported that UCH-L1 can discriminate between individuals with positive and negative CT scan findings [21]. Other promising biomarkers under active investigation for TBI diagnosis include NfL and S100B [21].

Ocular manifestations of TBI are among the most frequently reported complications. Orbital fractures occur in 18–67% of TBI cases, with the rate varying according to injury mechanism and severity [22]. Dry eyes and ocular surface disease were reported in 37% [23]. Photosensitivity affects 50% of individuals with TBI, negatively impacting daily activities [24]. Pupillary abnormalities were reported in 60% and 88% of TBI cases [25]. Structural damage of retinal layers has been documented in 30% of TBI cases, reflecting retrograde neurodegeneration of the visual pathway [26].

Given that the eyes are housed within the skull, TBI may damage them, affecting their structure and function [27,28]. This raises the possibility of identifying biomarkers to detect TBI of different severities, monitor, and assess recovery. A biomarker is a characteristic that can be objectively measured and evaluated as an indicator of normal biological or pathogenic processes, or of pharmacological responses to an exposure or intervention, including therapeutic engagement and recovery [29]. The current work aims to review ocular manifestations of TBI and assess whether these changes could serve as biomarkers for detection, monitoring, and recovery. The review excluded oculomotor dysfunction as it was recently reviewed elsewhere [30,31]. 

## 2. Methods

A search was conducted on the impact of TBI on the orbit, dry eyes and the ocular surface, pupillary response, and retina, as well as on a common TBI symptom: photosensitivity. Databases used include PubMed, Scopus, and Google Scholar, using relevant terms for each section, with the following representative search combinations. For orbital involvement, terms included [TBI OR traumatic brain injury OR concussion] AND [orbital fractures]. For ocular surface and dry eye disease, terms included [TBI OR traumatic brain injury OR concussion] AND [ocular surface disease OR dry eye disease OR ocular pain OR tear film OR corneal nerve]. For photosensitivity, terms included [TBI OR traumatic brain injury OR concussion] AND [photosensitivity OR photophobia]. For pupillary response, terms included [TBI OR traumatic brain injury OR concussion] AND [Pupillary response OR smartphone application]. For retinal changes, terms included [TBI OR traumatic brain injury OR concussion] AND [optical coherence tomography OR retinal thickness OR OCTA].

Inclusion criteria included published scientific articles in English, including retrospective and prospective observational studies. Conference abstracts were included when primary research was limited, while case reports were excluded. Articles were screened and included within the following publication years: orbit (January 2010–December 2025), dry eyes and the ocular surface (August 2000–June 2026), photosensitivity (August 2012–February 2024), pupillary response and smartphone applications (September 2014–April 2025), and changes in retinal characteristics (August 2017–September 2025). These date ranges reflect the availability of relevant literature meeting the inclusion criteria, with broader ranges for topics with a longer-established evidence base and narrower ranges for more recently emerging areas of research. The initial search yielded 404 articles, whose abstracts were screened. After excluding unrelated articles, 80 research papers were assessed; 37 were identified as duplicates across the databases and removed, resulting in 43 articles included. The last search was conducted in June 2026.

Additionally, the review was prepared with consideration of the SANRA quality criteria [32]. 

## 3. Results

### 3.1. Molecular Mechanisms of TBI-Associated Damage to the Visual System

The mechanisms underlying ocular manifestations in TBI are multifactorial and linked to the primary insult and neuroinflammatory processes of secondary injury. Oxidative stress, mitochondrial dysfunction, and apoptosis are among the molecular mechanisms involved and are reviewed briefly below.

#### 3.1.1. Neuroinflammation

Neuroinflammation is a critical pathological consequence of primary insult and principal driver of secondary injury. Innate immune activation in TBI begins within minutes of the primary insult and is driven by the release of DAMPs, including high-mobility group box 1 (HMGB1), uric acid, adenosine triphosphate (ATP), RNA, DNA, S100 calcium-binding protein B (S-100B), and interleukin-1α, from cells undergoing nonapoptotic death. These DAMPs activate pattern recognition receptors, including toll-like receptors (TLRs) and the receptor for advanced glycation end products (RAGE), on microglia and astrocytes, which release inflammatory cytokines, initiating a sterile immune reaction in an attempt to restore homeostasis [33]. This enhances the recruitment of monocytes, lymphocytes, and neutrophils across the BBB [34]. Furthermore, the NLRP3 inflammasome, a multiprotein complex found in microglia, plays a critical role in amplifying the inflammatory cascade [34].

Microglia have two inflammatory states. In their neuroprotective state, microglia prevent neuronal injury, remove cellular debris, and release neurotrophic factors and anti-inflammatory cytokines. However, a disinhibited and highly reactive microglial activation state results in a detrimental role, leading to the release of cytotoxic mediators and reactive oxygen species (ROS) that contribute to neuronal dysfunction, neurodegenerative disease, and cellular death [35].

**Figure 1:**
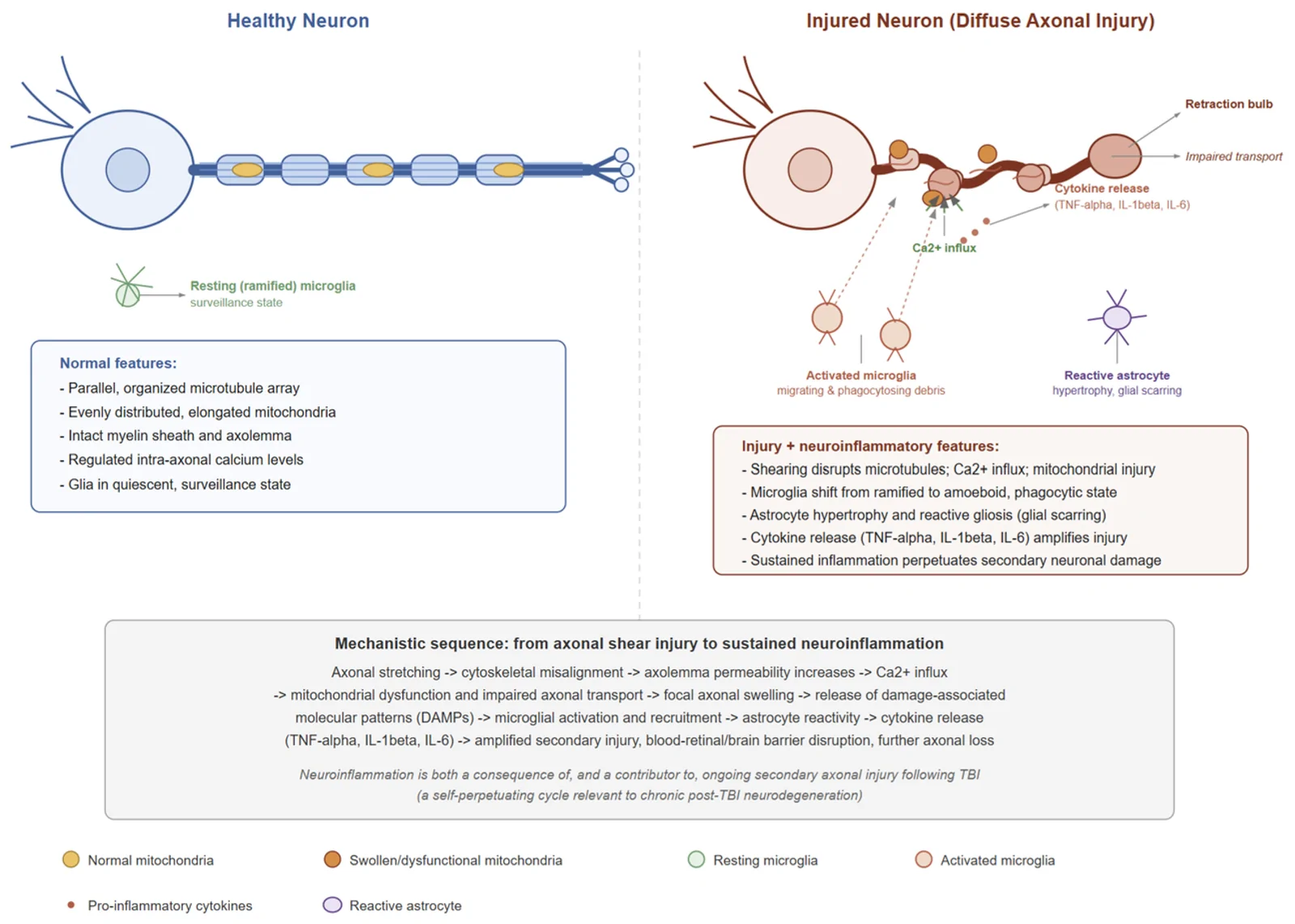
A representative illustration of neuroinflammation after TBI.

Astrocytes, the most abundant glial cells in the central nervous system, provide nutrients and metabolic support, recycle neurotransmitters, regulate blood flow, and, together with pericytes, maintain the integrity of the BBB [36]. Following TBI, astrocytes surrounding the lesion undergo hypertrophic reactive astrogliosis, releasing GFAP, S100B, and cytokines to recruit microglia and peripheral immune cells. Astrocytes also proliferate and intertwine to form an astroglial scar to control the spread of inflammation (Figure 1) [37].

The precise roles of peripheral immune cells, including monocytes and macrophages, in TBI pathogenesis are not completely understood. However, it has been suggested that they are among the first cells to die after injury and may be early sources of alarmins and ROS [33].

Within the visual system, neuroinflammation occurs at different levels, including the ocular surface, the retina, optic nerve, optic tract, lateral geniculate nucleus, and visual cortex.

On the ocular surface, due to TBI, the vicious cycle of dry eye disease, accompanied by upregulation of inflammatory cytokines, recruitment of immune cells, and sensitization of trigeminal afferents, and disruption of the lacrimal functional unit, may aggravate dry eye symptoms and perpetuate neuroinflammatory damage [38]. Supporting this, a proteomic analysis of tear fluid in individuals with traumatic vegetative state following TBI has identified alterations in proteins associated with inflammatory and tissue repair pathways, reflecting an active neuroinflammatory and reparative response at the ocular surface [39,40].

In the retina, three main types of glial cells contribute to retinal homeostasis: Müller cells, astrocytes, and microglia [41]. TBI induces upregulation of several neuroinflammatory, apoptotic, and neurodegenerative biomarkers. These include GFAP, ionized calcium-binding adapter molecule 1 (IBA1), cluster of differentiation 68 (CD68), phosphorylated tau, caspase-3, complement C3 and others [42]. These markers collectively reflect activation of Müller cells, astrocytes, and microglia, in addition to complement-mediated synaptic elimination, apoptotic signaling, and progressive neurodegeneration within the retina [42]. Sustained and unresolved inflammation eventually leads to the breakdown of the blood-retinal barrier (BRB), further compromising retinal integrity [41].

These neuroinflammatory changes extend beyond the retina to the optic nerve and optic tract, where axonal degeneration accompanied by neuroinflammation and astrocytosis has been documented within 7 days of injury, with microglial morphological changes detectable as early as 24 h post-injury and increased GFAP expression in the optic tract, lateral geniculate nucleus (LGN), and superior colliculus [43].

Molecular changes have been identified in ocular fluids. In repetitive TBI, changes in NfL and total tau (tTau) have been found in vitreous humor. Vitreous tTau levels correlate with those in brain tissue, suggesting that these biomarkers may reflect axonal damage and neurodegeneration that occur in the brain due to repetitive TBI [44].

#### 3.1.2. Oxidative Stress and Mitochondrial Dysfunction

ROS are free radicals that contain unpaired electrons, with peroxides produced under normal physiological conditions mainly by mitochondria as byproducts of ATP synthesis and metabolism [45]. Healthy mitochondria maintain redox homeostasis through defense mechanisms, including the thioredoxin system, regulation of the mitochondrial permeability transition pore (mPTP), and antioxidant enzymes such as vitamins E and C, superoxide dismutase 2 (SOD2), and glutathione peroxidase 1 (GPx1).

Following TBI, breakdown of the BBB and BRB allows infiltration of peripheral immune cells, which, together with microglia, release large amounts of ROS and inflammatory cytokines. Elevated ROS damage the mitochondrial membrane, impair electron transport chain function, trigger pathological opening of the mitochondrial permeability transition pore (mPTP), and cause oxidative modifications to mitochondrial DNA, collectively leading to progressive mitochondrial dysfunction. During this process, mitochondria swell and become sites of ROS-induced damage, and the resulting ROS damage nuclear DNA [46,47].

Within the visual system, retinal ganglion cells (RGCs) have high metabolic demands, necessitating high mitochondrial density. While initial thickening of the RNFL reflects acute axonal edema and neuroinflammation, elevated ROS drive lipid peroxidation, and mitochondrial dysfunction contributes to energy depletion, leading to progressive thinning of the RNFL and ganglion cell layer (GCL). Furthermore, ROS-mediated degradation of tight junction proteins disrupts the BRB, facilitating infiltration of peripheral immune cells and further amplifying retinal neuroinflammation and neurodegeneration [46].

Under normal conditions, the ocular surface is equipped with a complex and regulated antioxidant system to defend against exogenous sources of ROS, including ultraviolet radiation, tobacco smoke, and air pollution [48]. Following TBI, ROS are generated by injured cells, infiltrating neutrophils and activated macrophages, damaging corneal epithelial cells and sensitizing corneal nerve terminals, contributing to ocular surface inflammation, reduced corneal sensitivity and dry eye disease [39,40].

Corneal nerves have high metabolic demands, necessitating high mitochondrial densities within nerve terminals. Oxidative stress and mitochondrial dysfunction within corneal nerve terminals are among the pathological pathways that contribute to corneal nerve degeneration in peripheral neuropathies, though direct evidence in the context of TBI remains to be established [49,50].

#### 3.1.3. Apoptosis

Neuroinflammation, oxidative stress, mitochondrial dysfunction, and excitotoxicity contribute to regulated cellular death (RCD). RCD is programmed cell death that eliminates damaged cells, unlike accidental death from direct injury. One form of RCD is apoptosis, which is characterized by cell shrinkage, nuclear condensation, DNA fragmentation, and the formation of apoptotic cell bodies [51]. Apoptosis is executed by caspases via intrinsic and extrinsic pathways. The intrinsic pathway is initiated in response to intracellular stress, including toxic insult, DNA damage, high levels of ROS, and mitochondrial calcium overload. This activates pro-apoptotic proteins, which increase mitochondrial outer membrane permeability, releasing cytochrome c into the cytosol. The released cytochrome c contributes to the assembly of the apoptosome by binding to apoptosis protease activating factor-1, which activates caspase-9, which subsequently cleaves and activates executioner caspase-3 to initiate the apoptotic cascade [51]. Apoptosis can also proceed independently via apoptosis-inducing factor (AIF), which translocates from mitochondria to the nucleus to drive chromatin condensation and DNA fragmentation [52].

The extrinsic pathway is initiated by tissue injury and is triggered by the binding of extracellular ligands, such as TNF-α, to transmembrane death receptors, forming a death-inducing signaling complex. This activates caspase-8, which subsequently activates executioner caspase-3, leading to apoptosis [51]. An animal model study showed that caspase-3 activation occurs in severe TBI and repetitive mild TBI. Caspase-3 was present in the nuclei, perinuclearly, or on the ganglion cell layer, suggesting widespread apoptosis in the retina [53].

#### 3.1.4. Molecular Biomarkers in Ocular Biofluids

In the tear film, preliminary data suggest that CGRP levels are elevated following TBI and decline after five to six months [54]. Proteomic analysis in traumatic vegetative state, a complication of TBI, revealed that 57 proteins were upregulated and 15 proteins were downregulated [40]. The authors subsequently verified the levels of 5 upregulated proteins, including S100 calcium-binding protein A7 (S100A7), glutathione S-transferase P (GSTP1), complement factor H (CFH), kininogen 1 (KNG1), and alpha-1-acid glycoprotein 1 (ORM1), in addition to 2 downregulated proteins, including cystatin B (CTSB), protease serine 1 (PRSS1) [40].

In the vitreous humor, tTau levels have been measured. tTau is a microtubule-associated protein located in the axons of CNS neurons and serves as a structural element of the axonal cytoskeleton. A postmortem study found significant elevation of its levels in individuals with CTE and AD compared to controls. And significantly correlated with cortical brain tissue tTau levels [44]. Furthermore, the study found that vitreous NfL levels were higher at the low stage of CTE than at the no-CTE or high-CTE stages [44].

### 3.2. TBI-Associated Damage to the Visual System

#### 3.2.1. Orbital Fractures

The orbit is a delicate bony structure, a socket within the skull, formed by seven bones, housing the globe, extraocular muscles, orbital fat and the optic nerve. The frontal and sphenoid bones form the orbital roof, while the maxillary, zygomatic, and palatine bones form the orbital floor. The ethmoid, lacrimal, maxillary, and sphenoid bones form the medial wall, while the articulation of the zygomatic and sphenoid bones forms the lateral wall. Fractures of one or more of these bones can co-occur with TBI, with a significantly higher incidence observed in adult males [55,56]. The orbital floor is the most common site of orbital fractures due to its fragile structure, followed by the medial and lateral walls and the orbital roof [57].

**Figure 2:**
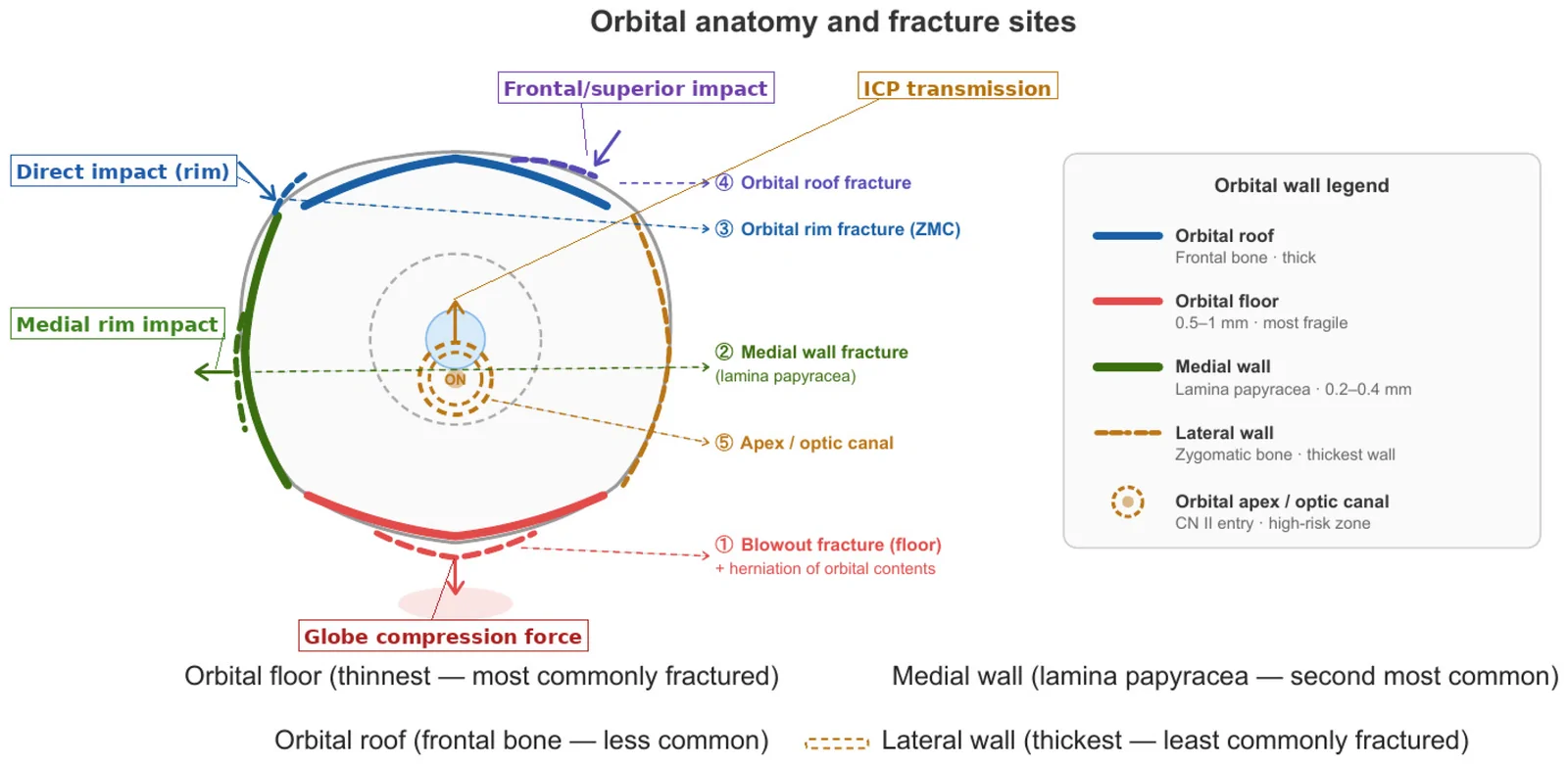
Illustration showing the sites of orbital fractures.

Orbital fractures result from direct mechanical impact or from the transmission of force from the craniofacial skeleton to the orbital bones, particularly the orbital floor and medial wall, which are vulnerable to damage [55,56]. Orbital fractures also occur concurrently with facial (maxillofacial) fractures since the majority of them result from midface trauma [57]. Types of orbital fractures include pan-facial fractures, Le Fort type 2 and 3 fractures [58,59,60]. A pan-facial fracture results from simultaneous fractures of the frontal bone, maxilla, zygomatic complex, nasoethmoid-orbital region, and mandible [59]. The Le Fort type 2 fracture is a facial fracture in the bridge of the nose and extends obliquely through the orbit and inferior orbital rims. Le Fort type 3 fracture passes through the nasofrontal area across the medial, posterior, and lateral orbital walls, zygomatic arch, and pterygoid plates [61]. Figure 2 shows the most common locations of orbital fractures.

Table 1 presents study designs, participant numbers, and main findings of the reviewed studies. A retrospective report reviewed data from 125,000 patients with ocular trauma and found that 65% of those with orbital fractures had associated TBI [62]. The report noted that the most common orbital fracture is a closed orbital floor fracture, most likely associated with mild TBI, followed by floor fractures without intracranial injury, and then open floor fractures. Orbital roof fractures are rare and typically associated with severe TBI, visual pathway injury and leakage of cerebrospinal fluid [62].

A study found that 67% of patients with facial fractures had concurrent head injuries. Specifically, zygomatic-maxillary complex fractures were found in 19% of cases, followed by maxilla fractures in 18% of cases and frontal bone fractures in 14% of cases, all of which are related to the orbit [22]. This is consistent with another report, which found a high prevalence and association with TBI in those with frontal bone and zygomatic bone fractures, and naso-orbito-ethmoid fractures [63].

Other severe facial fractures, including pan-facial fractures and Le Fort type 2 and 3 fractures, have been associated with neurosurgical intervention in moderate TBI [60]. Other reports showed that the severity of these fractures is linked with TBI severity [58,68].

##### Neuroimaging

One retrospective study reviewed facial bone CT scans to identify those who are at risk of concomitant TBI among those whose chief complaint was orbital fracture. It found that superior orbital wall fracture and associated frontal bone fracture were independent risk factors for intracranial injury even in patients without typical TBI symptoms [64].

Another study utilizing CT and MRI found that 69% of orbital fractures occurred in those with TBI, concluding that orbital fractures should not be viewed as isolated facial injuries but as a clinical marker for potential multisystem trauma [65].

It has been reported that orbital fractures are associated with reduced gray and white matter density in various brain regions, as imaged by MRI, compared with healthy individuals [66]. In gray matter, reductions were found in the right superior temporal gyrus, right middle temporal gyrus, bilateral inferior frontal orbital gyrus, left superior temporal gyrus, anterior cingulate and paracingulate gyrus, and in the right insula. In white matter, reductions were also found in the right parahippocampal gyrus [66].

Another study utilized the fractional amplitude of low-frequency fluctuation (fALFF) method and found that those with orbital fractures had lower fALFF values in brain regions including the left anterior cingulate gyrus and the right superior temporal gyrus [67].

#### 3.2.2. Ocular Surface and Dry Eye Disease

The ocular surface comprises several interconnected structures, including the eyelids, meibomian glands, lacrimal gland, tear film, conjunctiva, cornea, and associated microstructures such as corneal nerves. TBI has been shown to affect the ocular surface and disrupt its homeostasis, contributing to the development of dry eye disease and ocular surface pain [23,38].

Dry eye disease (DED) is a symptomatic, multifactorial condition characterized by loss of tear-film homeostasis and inflammation of the ocular surface, with neurosensory abnormalities. Disruption of ocular surface homeostasis in TBI could arise from damage to the ophthalmic branches of the trigeminal nerve that innervate the lacrimal gland and the cornea. This may reduce corneal sensitivity and impair reflex and basal tear secretion, resulting in ocular surface inflammation. Other possible mechanisms include altered blinking rate, facial palsy, and autonomic dysfunction [23]. Furthermore, photosensitivity is considered one of the DED symptoms [69] and it has been suggested that it shares a similar light-pain pathway, namely the trigeminothalamic pathway, due to sensitization, with migraine and TBI [39].

Table 2 presents study designs, participant numbers, and main findings of the reviewed studies. A study investigated ocular surface staining, the Ocular Surface Disease Index (OSDI) score, tear break-up time (TBUT), tear osmolarity, tear production, meibomian gland dysfunction (MGD), and corneal sensitivity, and assessed cranial nerve palsy in 53 male individuals with varying severities of TBI [38]. The authors reported that individuals with TBI had increased ocular surface staining and higher OSDI scores, which worsened over time, compared with healthy individuals. As reflected in OSDI subdomains, the most commonly reported symptoms include photophobia, blurred vision, difficulty reading, and discomfort in low-humidity environments. Other measured parameters, including TBUT and reduced corneal sensitivity, showed marginal changes without statistical significance, while a small number had MGD and facial nerve palsy. The authors also indicated that dry eye measures were independent of TBI severity [38].

A retrospective study investigated the incidence of anterior segment disease in 160 patients with unreported severity of TBI, including dry eye disease, pinguecula, blepharitis, chalazion and hordeolum. The authors indicated that DED and blepharitis occur in 15.5% and 18% of individuals with TBI, respectively. Other conditions, including MGD, corneal abrasion, superficial epithelial keratitis, and lid lesions, occur less frequently [70,71].

A retrospective study investigated the relationship between dry eye disease, ocular surface pain, TBI and its comorbidities, including chronic pain syndrome, headache, depression, and post-traumatic stress disorder, in a cohort of 125,000 American veterans, using hierarchical cluster analysis. The authors found that DED, ocular pain and tear film dysfunction occurred more frequently in individuals with TBI compared to healthy individuals [23]. The study also demonstrated that multiple conditions associated with late TBI sequelae were clustered with dry eye disease. The first cluster comprised dry eye disease, central pain syndrome, headache, and late effects of nervous system injury. The second cluster included visual discomfort, joint dysfunctions, and headaches. The third cluster included pain in or around the eye, irritable bowel syndrome and TBI [23]. Additional conditions, including migraine, anxiety, depression, and sleep disturbance, were also found to cluster with tear film insufficiency. Furthermore, ocular pain was strongly associated with TBI, and long-term late effects on the nervous system increase the risk of DED by 1.7 times.

#### 3.2.3. Photosensitivity

Photosensitivity or photophobia is defined as an aversion to a light source that normally does not provoke visual discomfort in the absence of an identifiable ocular cause. It is a common chronic symptom in those with TBI and a frequent complaint after head injury [72]. The photosensitivity mechanism is complex, and proposed mechanisms include trigeminothalamic sensitization; neuroinflammation; disruption of the pupillary light reflex pathway; damage or sensitization of intrinsically photosensitive retinal ganglion cells (ipRGCs) and their thalamic projections [39,73,74,75]. ipRGCs are non-image-forming retinal ganglion cells (RGCs) and key components of the pupillary light reflex circuit, playing an essential role in regulating circadian rhythms and the sleep–wake cycle by expressing melanopsin. They exhibit peak sensitivity to blue light at a wavelength of 480 nm and continue firing for several seconds after the light stimulus is removed [76]. According to a recent meta-analysis, photosensitivity affects 30% of individuals with TBI with varying severity, particularly within the first week of injury. This proportion declines to 19% between the first week and the first month. Further, it drops to 14% between the first and third months, although it can persist for up to 12 months in some cases, with a significant change in quality of life [24].

Table 3 presents study designs, participant numbers, and main findings of the reviewed studies. In mild TBI, half of the individuals complained of photosensitivity based on a report using the Neurobehavioural Symptoms Inventory (NSI) questionnaire [73]. This is consistent with a recent report indicating that approximately 42% experienced persistent sensitivity to light and noise, as measured by the Rivermead Post-concussion Symptoms Questionnaire, which was associated with reduced quality of life for up to 12 months [77].

Another study used an ocular photosensitivity analyzer (a device that delivers graded light stimuli and records when participants report discomfort), along with measuring pressure pain thresholds and TBI-related comorbidities, including chronic pain, post-traumatic stress disorder, post-concussive syndrome, depression, and insomnia in symptomatic and asymptomatic individuals with TBI of different severity and in healthy individuals [72]. Although pressure pain thresholds did not differ between the groups, those with TBI exhibited lower thresholds of photosensitivity and reported higher scores on chronic pain assessment surveys. Within the TBI group, symptomatic individuals demonstrated greater photosensitivity and reported more severe chronic pain compared to asymptomatic individuals. The study also found a strong negative correlation between the photosensitivity threshold and overall chronic pain. This relationship was attributed to altered activity and reduced connectivity within the central nervous system [72].

The severity of photosensitivity has also been associated with the number of mild TBIs and comorbidities, including post-traumatic stress disorder (PTSD), poor sleep quality, and elevated anxiety, as observed in post-9/11 veterans with mild TBI. Using the NSI questionnaire, photosensitivity was greater in veterans with mild TBI compared to their counterparts without TBI. It was further intensified when TBI co-occurred with PTSD and sleep disturbances or if they had repetitive mild TBI [73].

A study used a flickering blue-and-red light stimulus to isolate ipRGC response and to differentiate between those with mild TBI and photophobia and healthy individuals but found no difference. Instead, greater variability was observed in blue-light pupillary response, suggesting heterogeneous degrees of ipRGC dysfunction within individuals with mild TBI [76]. Another study that used similar methods and suggested that neither blue nor red light stimulus type isolates ipRGC-driven components of the pupil response [79].

Another study utilized a binocular pupillometer (Neuroptics DP-2000, Neuroptics Inc., Irvine, CA, USA) with white, red, and blue light stimuli. The study found significant differences between photosensitive and non-photosensitive individuals, with and without mild TBI, across various pupillary measures in white-red conditions. These included a larger pupil diameter throughout the dynamic pupillary light response profile and faster dilation velocity and faster times to reach 50% (T50) and 75% (T75) of pupillary re-dilation. In blue light conditions, a smaller 6 s post-stimulus pupil diameter (6SPSD) was found in photosensitive mild TBI individuals compared to non-photosensitive individuals [78].

A retrospective study investigated changes in pupillometric parameters (Neuroptics DP-2000, Neuroptics Inc., Irvine, CA, USA) in relation to photosensitivity in children with concussion (mean age of 13.8 ± 2.6 years) compared to normative data. Photosensitivity did not impact any of the pupillary metrics. The study found faster dilation and constriction velocities, along with faster 75% recovery time [80].

#### 3.2.4. Pupillary Abnormalities

The pupillary light reflex is initiated by the activation of RGCs that transmit signals through the optic nerve, optic chiasm, and optic tract to the pretectal area in the midbrain. From there, secondary fibers project bilaterally to the Edinger-Westphal nuclei, which contain preganglionic parasympathetic neurons. The preganglionic neurons generate motor signals that travel via the oculomotor nerve to the ciliary ganglion and synapse on postganglionic neurons, which innervate the sphincter pupillae muscle, resulting in pupillary constriction. In contrast, sympathetic signals originate in the hypothalamus and descend through the brainstem and the spinal cord to synapse in the ciliospinal center of Budge. Preganglionic sympathetic neurons then project to the superior cervical ganglion, where they form synapses. Postganglionic fibers travel along the internal carotid artery and through the long ciliary nerve to innervate the dilator pupillae muscle.

The clinical assessment of the pupillary light reflex represents the health status of the pupillary pathway and the autonomic nervous system. In those with TBI, disruption of the neuronal circuit controlling pupillary function results in abnormal pupil diameter, shape, and response. Hence, pupil evaluation plays a significant role in assessing patients with TBI and is an essential part of the GCS to assess neurological deterioration, often termed neuro-worsening. Pupillary abnormalities in mild and moderate TBI result from an imbalance between the sympathetic and parasympathetic systems, which regulate pupillary tone [81]. In severe TBI, fixed pupils result from increased intracranial pressure, leading to uncal herniation and compression of the parasympathetic fibers of the oculomotor nerve [82].

Traditionally, a penlight with a manual gauge is used to assess pupil size and reactivity (e.g., brisk, sluggish, fixed), and the interpretation of the results is subjective and varies among practitioners. Recently, quantitative pupillometry metrics using infrared technology have been proposed as an alternative tool for assessing the pupillary response [81,83,84]. The device generates a composite score known as the neurological pupil index (NPi), calculated from pupil size, latency, diameter, percentage change, and relative constriction and dilation velocities. NPi scores range from 0, indicating no pupillary reaction, to 5, indicating a normal response. Values equal to or below 3 are considered abnormal [84]. Another generation of pupillometers has been introduced (PLR-3000, Neuroptics, Irvine, CA, USA). The device provides detailed quantitative pupillometric measurements for both eyes, including initial and minimum pupil diameters, constriction latency, mean and maximum constriction velocities, average dilation velocity, peak dilation velocity, percent change, graph trace, and T75 recovery [85]. Furthermore, smartphone applications have been proposed for evaluating pupillary responses [86,87,88]. PupilScreen is an application that uses the smartphone’s flashlight and camera to stimulate the patient’s pupils and record pupil diameter and centration. The application then generates a two-dimensional curve that represents the relationship between time (in seconds) and changes in pupil diameter (in pixels), with a median error of 0.36 mm [89]. Another iPhone-based application named Reflex-Pro analyzer generates a photic flash stimulus with a length of 1 millisecond and 10 lumens, set to 70% power, and records dynamic pupillary reaction at 30 Hz (30 frames per second) [90].

##### NPi-200 Pupillometer

Table 4 shows study designs, participant numbers, and main findings of the reviewed studies. A study examined whether NPi generated by the NPi-200 pupillometer (Neuroptics Inc., Irvine, CA, USA) could predict neurological deterioration, as assessed by GCS, within the first 24 h in 95 individuals with varying severity of TBI [81]. The authors reported that 35 individuals experienced neuro-worsening within 24 h compared with baseline, and 66% had an initial NPi decline to below 3. In contrast, three patients had scores below 3 without neuro-worsening. The study also found a significant association between an NPi score ≤ 3 and neuro-worsening, with high specificity (92%) and low sensitivity (51%).

Another report showed that NPi scores below 3 on admission could predict unfavorable neurological outcomes in a cohort of individuals with different severities of TBI [91]. Another report demonstrated that a low NPi score is useful for triaging patients with moderate to severe TBI who require emergent intervention for a closed head injury, especially when brain imaging does not show a direct sign of brain herniation, midline shift, or increased intracranial pressure [92].

A study used a similar pupillometry model to examine associations among NPi score, loss of consciousness, subsequent TBI outcome, and various pupil parameters. These included initial pupil size, anisocoria > 1 mm, the latency of constriction, average and maximum constriction velocity, average dilation velocity, minimum pupil diameter at peak constriction, and the percentage of change [84]. The authors found a significant association between decreased dilation velocity and loss of consciousness. Also, NPI scores were associated with poor functional outcomes, increased intracranial pressure, and a higher likelihood of emergency procedures. In contrast, faster constriction velocity was associated with need for emergency intervention and better 3-month functional outcomes but not with loss of consciousness. The majority of the population in this study had moderate to severe TBI and a few cases of mild TBI [84].

A study of asymptomatic high school football athletes (mean age 17.2 ± 0.6 years) examined pupillary metrics generated by the NPi-200 in relation to High-acceleration Head Impact (HHI: linear acceleration > 95 g and rotational acceleration > 3760 rad/m^2^) [93]. HHI was measured using a helmet-mounted sensor, with assessments conducted pre-season, mid-season, immediately after the injury and at the end of the season. Significant reductions were found in dilation velocity, percent change in pupil diameter, and maximum constriction velocity following the HHI compared with the athletes’ own values at the midpoint, as well as changes in constriction and maximum constriction velocities over the course of the season, without changes in neurocognitive functions. Athletes who self-reported higher event intensity, despite recorded forces below the HHI threshold, also showed significant reductions in the same metrics [93].

##### PLR-3000 Pupillometer

A recent study compared pupillary metrics in 12 female hockey athletes with a history of concussion that occurred between two weeks and one year, and athletes without [85]. No significant differences were found. Only two individuals who had concussions within the previous three months had a trend of slower constriction latency, average constriction velocity, average dilation velocity and peak dilation velocity compared to those with no history of concussions or who had concussions for more than three months.

Another report investigated whether the quantitative pupillary metrics could reflect the severity of concussion symptoms in 162 participants with mild TB (GCS 13–15), assuming that those with acute symptoms would present early to the clinic. The report included participants with a recent injury (within two weeks) and those with an injury lasting more than two weeks [94]. The severity of the concussion symptoms was assessed using SCAT5 [11], and PCSS [12]. The authors found a significant correlation between anisocoria after light exposure and symptom severity. Additionally, significant reductions in average and maximum constriction velocities were observed in those who presented early to the clinic compared with those who presented later.

A recent study showed that differences in pupil diameter, maximum constriction velocity, and average dilation velocity can distinguish participants who returned to duty before or after 21 days in cadets with mild TBI [95]. Pupillary measurements were taken four times: preinjury baseline, 48 h after the injury, when the participants were symptom-free and resumed training, and when they were released for unrestricted activity. The authors reported that larger and faster pupillary constriction was associated with a shorter time to return to full activity, as measured preinjury and 48 h postinjury. A higher average dilation velocity was associated with return to duty only when the cadets were released for unrestricted activity.

##### Pediatric Settings

A report examined the association between concussion and the pupillary metrics and whether these metrics could identify those at increased risk for persistent symptoms in children aged 5–11 and 12–18 years old. Participants were assessed within 72 h post-head injury, after two weeks, and after three months, and compared with healthy individuals. Concussion symptoms, postural stability, and neuropsychological function were also assessed. In children aged 5–11 years, the symptoms were significantly worse in the concussion group during the initial visit, while pupillary metrics showed no differences [96]. Children aged between 12 and 18 years showed higher symptom scores, lower cognitive performance and postural stability, and smaller minimum pupil diameter (greater pupil constriction) during constriction in the left eye only, compared with healthy individuals at the initial visit. Postural stability and cognitive function were significantly improved at the third visit, with noticeable variations in pupillary metrics [96].

Another study assessed pupillary metrics in athletes aged between 12 and 18 years with concussions, with measurements taken within 28 days post-head injury. All pupillary metrics were significantly increased except for latency. Conducting a sub-analysis within 7 days of the injury revealed no significant differences in average dilation velocity or time to 75% recovery, while the significance remained for other metrics. Interestingly, a longer T75 was noted in females compared to males with concussions [97].

##### Smartphone Applications

Using PupilScreen, 84 curves from 48 healthy participants and 36 curves from six participants with severe TBI were generated from both eyes. Then a random selection of curves from both groups was interpreted as normal or abnormal by six resident neurologists and five nurses working in a neuro-intensive care unit. The ability to determine abnormal values from the application was 93%, 91%, and 92% for physicians, nurses, and both groups, respectively [87]. Another study examined the validity of the same application by comparing it with the pupillary index derived from the NPi-200 pupillometer in healthy individuals and those with severe TBI [86]. PupilScreen demonstrated higher accuracy, sensitivity, area under the curve, and F1 score in differentiating healthy individuals from those with severe TBI compared to the NPi-score.

Following this, a recent study examined the accuracy of a machine-learning approach that combined latency, percentage change, maximum and minimum pupil diameters, mean and maximum constriction velocities, and mean dilation velocity, as generated by PupilScreen, in identifying male individuals with acute sport-related concussions [98]. Baseline measurements were taken from 93 football players and repeated for those who had sport-related concussions. Symptoms and symptom severity scores were assessed using the Sport Concussion Assessment Tool 6 (SCAT6). Data from 11 injured players were augmented using the synthetic minority oversampling technique and then evaluated using multiple machine learning models. The most effective combination was generated by the random forest algorithm. It included latency, maximum and minimum diameter, mean and maximum constriction velocity, producing 91% accuracy, 98% sensitivity, 84% specificity, 0.91 area under the curve, and an F score of 92% in differentiating between baseline and the parameters after the head injury [98].

Another study used the Reflex-Pro PLR analyzer to compare pupillary parameters between healthy individuals and athletes who have had sport-related concussion, with persistent concussion symptoms and photophobia, for a duration of at least one year [90]. Mean constriction velocity, maximum constriction velocity, average diameter, and maximum and minimum diameters significantly decreased in those with concussion, while rapid re-dilation was found in those with photosensitivity compared to those without, within the TBI group.

#### 3.2.5. The Retina

The retinal nerve fiber layer (RNFL) comprises unmyelinated axons of retinal ganglion cells, supported by glial cells, and converges to form the optic nerve, which transmits electrical signals to the brain. Structural changes in retinal layers in TBI arise from primary mechanical injury and secondary neurodegenerative processes. Acute thickening of the retinal layers occurs initially and results from axonal swelling, neuroinflammation, and microglial activation. Following this, progressive thinning ensues through retrograde transsynaptic degeneration and RGC apoptosis [99]. Furthermore, reduction in macular microvascular perfusion also occurs and has been proposed to reflect the cerebrovascular disruption after TBI, since the eye originates from the brain during the embryonic period [100]. Moreover, damage also extends to the Henle fiber layer, bipolar cells, and the photoreceptors, possibly due to neuroinflammation and Müller cell activation [101].

Table 5 shows study designs, participant numbers, and main findings of the reviewed studies. A longitudinal study utilizing spectral-domain optical coherence tomography (OCT) assessed RNFL and GCL thickness in individuals with mild TBI and also evaluated visual field, contrast sensitivity, and cognitive functions. It found thicker RNFL thickness at baseline. Subsequently, RNFL thickness decreased significantly over time compared with healthy individuals, accompanied by declines in visual-cognitive functions [99]. A separate study assessed RNFL thickness and visual field at 7 days, 3 months, 6 months, and 1 year post-mild TBI [26]. At 3 months post-mild TBI, 31% of participants showed reduced RNFL thickness, with further thinning at 6 months and remained stable up to 1 year. This was accompanied by a reduction in visual fields at 3 months and persisted at a similar level at 6 months and 1 year. RNFL thinning was strongly correlated with visual field defects at every point [26].

A recent study found no difference in retinal layer thickness or volume between individuals with mild or moderate TBI and healthy individuals. After excluding female participants, male participants had reduced thickness and volume of 1 mm in the central ganglion cell-inner plexiform layer (GC-IPL), along with increased thickness and volume within 3 mm and 6 mm of the outer plexiform layer (OPL). Changes in GC-IPL and OPL were correlated with the time of injury [102].

Another study categorized retinal changes among individuals with single or multiple TBIs of varying severity and assessed their relationship to brain imaging and visual field defects [103]. No significant difference in the RNFL between those with single or multiple TBIs, consistent with previous work [108]. Using test–retest data in healthy individuals and in individuals with glaucoma, pseudophakia, and diabetes, the authors define the limits of agreement for RNFL and GCL thickness. Based on this, half of the participants showed significant changes in the RNFL or GCL on longitudinal follow-up, with predominant multisectoral thinning and traumatic optic neuropathy associated with moderate-to-severe TBI. A small number of participants also developed homonymous GCL thinning, which corresponded to visual field defects or traumatic optic neuropathy. Greater RNFL thinning was significantly associated with CT findings and TBI severity, with fastest thinning observed in Marshall score II patients within the first 150 days [103].

A retrospective study assessed RNFL thickness in the peripapillary area, the macular RGC layer, and the subfoveal choroidal layer in patients with a history of mild TBI, with follow-up ranging from 3 to 35 years post-injury. The study demonstrated that those with chronic mild TBI had a mild form of indirect traumatic optic neuropathy, characterized by thinning of the subfoveal choroidal layer and the temporal sector of the peripapillary RNFL, corresponding with thinning in the macular GCL. This was accompanied by mild symptoms, including photophobia, blurred vision, flashes, and floaters [104].

Another study utilized OCT angiography (OCTA) to investigate the retinal and choroidal vasculature in mild and moderate TBI [100]. A significant reduction in the circularity of the foveal avascular zone (FAZ) was observed in both groups, accompanied by reductions in vessel and perfusion densities within 3.0 mm × 3.0 mm circles and rings. No significant differences in RNFL, GC-IPL, and choroidal thickness. Comparisons within the TBI group showed that those with PTSD had reduced FAZ circularity and vessel and perfusion densities in both macular areas (3.0 mm × 3.0 mm and 6.0 mm × 6.0 mm).

Individuals with moderate TBI showed a significant increase in the choroidal vascularity index and changes in macular OCTA parameters compared with those with mild TBI without loss of consciousness. Those with more than one TBI had a lower choroidal vascular index than those with one TBI (Table 5) [100].

Another study examined whether reduced retinal perfusion would precede and predict GCL neurodegeneration in those with moderate to severe TBI [105]. No significant differences in GCL thickness or retinal perfusion were observed when measured acutely within 14 days post-injury. At follow-up between two and six months, 43% of participants showed GCL thinning, which was correlated with reductions in macular retinal perfusion in the superficial vascular plexus. Other vascular plexuses (inner and deep) showed a weaker association.

A study investigated the global thickness and phase retardation of the Henle fiber layer using directional OCT and scanning laser polarimetry. The latter was used to assess the structural integrity of microtubules within photoreceptors. Photoreceptor and bipolar cell function was assessed using electroretinography (ERG) [101]. A significant increase in the thickness of the Henle fiber layer outside the central fovea was observed in those with multiple mild-to-moderate TBIs compared to healthy individuals, with no corresponding difference in phase retardation between groups. ERG showed a reduction in the a-wave in the light-adapted condition, suggesting impairment of off-cone bipolar cell responses. The amplitude of the b-wave was trended lower in TBIs, but the difference was not statistically significant.

##### Sports

In a sports setting, a study examined retinal structures in 16 Olympic boxers who were exposed to repetitive concussions using OCT at two visits over 18 months [106]. Macular thickening and changes in RNFL thickness were observed across most tested parameters at the second visit compared with baseline values. Compared with healthy individuals, these parameters were lower [106].

Another study investigated retinal layer thicknesses and visual function in athletes, including boxers, ice hockey players and football players [107]. RNFL thinning was observed in the athlete cohort compared with healthy individuals, driven by Boxers, who also had reduced ganglion cell complex (GCC) and poorer low-contrast sensitivity compared with healthy individuals. No changes in retinal layer thickness were observed in football players, while ice hockey players were excluded.

## 4. Discussion

The current review aims to assess ocular manifestations resulting from TBI and examine whether these manifestations could be used as biomarkers for detection, monitoring, and recovery. This includes orbital fractures, DED and ocular surface changes, molecular biomarkers in ocular fluids, photosensitivity, pupillary abnormalities, and retinal changes, in addition to associated molecular mechanisms. Table 6 summarizes these entities and shows their replication status.

Orbital fractures occur in 65–69% of TBI cases. Fracture severity tends to match TBI severity, as floor fractures and radiologically occult fractures often occur with mild TBI, while more complex fractures such as pan-facial or Le Fort fractures occur with severe TBI [62,65,68]. Complex fractures significantly damage the orbit and its contents, cause downward displacement of the globe, entrap the extraocular muscles within the bone fractures, lead to injury of the infraorbital and optic nerves, and cause cerebrospinal fluid leakage. While severe fractures alone do not add diagnostic value for TBI since patients are already recognized as having major trauma, subtle or hidden fractures could be more clinically useful, as they have been linked to reductions in white and gray matter and altered brain activity, and can be attributed to three possible mechanisms [66]. First, a concomitant TBI could have occurred, triggering neuroinflammation and glial activation, leading to progressive volume loss in gray and white matter. Another possibility is anterograde degeneration of the optic nerve, causing neurodegeneration along the visual pathway to the lateral geniculate nucleus and the visual cortex. Furthermore, the loss in volume could be due to the activation of the trigeminal pain pathway since trigeminal nerve damage projects pain signals to the insula, anterior cingulate, and orbital gyrus cortex [66,67]. Since the subtle fracture does not reflect ongoing biological processes, assessment of orbital fracture is best suited as a diagnostic indicator tool for occult mild TBI, rather than monitoring or predicting outcomes.

In dry eyes and the ocular surface, based on the reviewed studies, findings on DED prevalence are inconsistent, ranging from 15.5% to 37.5% [23,38,70,71]. This could be due to methodological differences. One study used established diagnostic criteria to diagnose DED [38], while other studies are retrospective and did not clarify how DED was diagnosed [23,70,71]. In a study, Cockerham et al. indicated that dry eye measures were independent of TBI severity [38]; however, this claim requires further studies since various tests can be combined to assess severity [109], and in their work, they relied only on the OSDI questionnaire, which reflects symptom burden [38]. The lack of association between DED and TBI severity could be due to the small sample size, as the subgroup severity was not statistically powered. Therefore, the conclusion here is questionable. DED of varying severity has been linked to changes in corneal nerve characteristics, inflammatory cytokines, and neuropeptides [110,111,112]. Individuals with TBI have more severe symptoms of DED, possibly due to trigeminal nerve sensitization and neurosensory abnormalities of the lacrimal gland, which may contribute to the inflammatory process in DED. However, direct evidence on structural damage to corneal nerves in TBI remains unexplored. Corneal nerves originate from the ophthalmic branch of the trigeminal nerve and consist mainly of polymodal nociceptors, mechanoreceptors, and thermoreceptors. They play a key role in ocular surface homeostasis by orchestrating the blink reflex, regulating neuroimmune responses, and promoting wound healing [113,114]. Trigeminal nerve sensitization can extend to corneal nerves and disrupt homeostasis. Assessment of corneal nerve morphology, together with tear film biochemistry, may help identify individuals with TBI of varying severity [115]. A substantial body of literature suggests that corneal nerve characteristics are altered (loss of fiber density and decreased length) in neurodegenerative disease and peripheral neuropathy [50,116]. Furthermore, applying deep learning and artificial intelligence can identify those with different neurological conditions from healthy individuals [117]. This represents a knowledge gap as there are no reports from those with TBI.

Similarly, tear film biochemistry studies have shown that several proteins and neuropeptides are either up- or downregulated in Alzheimer’s disease, Parkinson’s disease, multiple sclerosis, and migraine, including Tau protein, synuclein, oligoclonal bands, and CGRP [118]. These secretions are released from epithelial cells of the ocular surface and terminal ends of the subbasal nerve plexus. In a preliminary report, tear film CGRP showed that it could reflect the latency of TBI [54], while another study suggests that changes in S100A7, GSTP1, CFH, KNG1, ORM1, CTSB, and PRSS1 reflect wound healing and the activation of the inflammatory response in the traumatic vegetative state in TBI [40]. Given that more than 2000 proteins are present in the tear film, and tears can be collected non-invasively compared to other bodily fluids, it would be of interest to examine their behaviors in the tear film. This represents a knowledge gap; exploring it alongside changes in corneal nerves and corneal immune cells may help elucidate how dry eye disease and TBI are interlinked.

In another form of ocular fluid, a postmortem study examined molecular markers in vitreous humor in a complication of TBI, that is, CTE. The concordance in the elevation of tTau between the vitreous humor and the brain tissue supports the potential of vitreous humor as a non-invasive proxy for brain tau pathology following repetitive TBI [44]. tTau protein is a microtubule-associated protein located in the axons of CNS neurons and serves as a structural element of the axonal cytoskeleton [44]. Furthermore, vitreous NfL levels were higher at the low stage of CTE than at the no-CTE or high-CTE stages, suggesting that it would reflect the severity of CTE [44]. NfL is a structural protein found in neurons and represents the internal skeleton of the neurons. Neither has been measured in tears in TBI, which is less invasive to collect.

Returning to dry eye disease and as reflected by the OSDI subdomain, photophobia is a common symptom of those with TBI and DED [38]. Beyond this shared symptom, photosensitivity in TBI arises through a complex, multifactorial mechanism. In DED and TBI, photophobia has been attributed to sensitization of the trigeminal nerve, interruption of neuronal pathways, and decreased production of neurotransmitters or nerve growth factors caused by chronic inflammation affecting both peripheral and central pathways [38]. Sensitization is defined as an increased responsiveness of nociceptive neurons to normal input and/or to normally subthreshold inputs, due to inflammation and neuronal dysfunction [23] Aligning with this view is a recent review suggesting that dry eye disease, TBI, and migraines are comorbid, share the symptom photosensitivity, and may share a similar light-pain pathway, the trigeminothalamic pathway, due to sensitization [39]. The review suggested that altered gene expression and receptor activation of CGRP trigger an inflammatory cascade that leads to overstimulation of peripheral nociceptors and neuroplastic changes in recipient central neurons. This neuronal sensitization may result in unpleasant sensations and pain in response to normal light stimuli in individuals with dry eyes, TBI, and migraines. This mechanism warrants further investigation, for example, measuring CGRP concentration in tears from individuals with TBI with and without photosensitivity. Some supporting evidence already exists. It has been suggested that 18% of photosensitivity in individuals with dry eye disease is attributable to sensitization or abnormalities in the central nervous system, as determined after local anesthesia is applied to the ocular surface [119].

Photosensitivity affects 30– 50% of individuals with varying TBI severities during the first week and drops to 14% after three months [24,73,77]. The steep decline in prevalence suggests that photosensitivity could be a potential biomarker of recovery, potentially reflecting the resolution of acute neuroinflammation and trigeminal nerve sensitization after the injury, or could reflect a compensatory neural mechanism. Also, photosensitivity can persist for up to 12 months in some cases, possibly due to chronic central sensitization of the trigeminothalamic pathway rather than acute injury [39]. A significant limitation is that the cited source did not stratify the prevalence by injury severity, a limitation acknowledged by the original authors [24]. Furthermore, all these findings were obtained from questionnaires that relied on subjective symptoms.

A more objective study measured photosensitivity threshold using a photosensitivity analyzer in relation to chronic pain in TBI with varying severity [72]. In this work, the lower photosensitivity threshold despite unchanged pain threshold in those with high pain score suggests that photosensitivity results from changes in the light-processing pathway rather than from generalized pain hypersensitivity. At the same time, within the TBI group, the severity of photosensitivity was greater in symptomatic compared to asymptomatic and strongly correlated with overall chronic pain severity, suggesting that photosensitivity and chronic pain may still share a common underlying driver, such as central sensitization, even if they are not mechanistically identical [72]. Supporting these findings is a recent study that utilized functional magnetic resonance imaging (fMRI) and showed greater activation of several brainstem and cortical regions in response to light stimuli in individuals with chronic ocular surface pain and photosensitivity, compared with healthy individuals [120]. These regions included the spinal trigeminal nucleus, the primary somatosensory cortex, and the insular and anterior mid-cingulate cortices [120]. Investigating whether similar patterns are present in individuals with TBI may help to elucidate the underlying mechanism of photosensitivity in TBI.

Greater photosensitivity could also distinguish those with repetitive mild TBI from those with a single mild TBI, suggesting a cumulative effect. Furthermore, having a psychological disorder, e.g., PTSD comorbid with TBI, was associated with greater photosensitivity compared to those without, using the NSI questionnaire [73]. In this work, it has been suggested that an alternative mechanism in those with TBI and PTSD may occur, suggesting that a hyperarousal state may drive photosensitivity symptoms and potentially result from dysregulation of widespread cortical functional networks involved in pain perception and light processing [73]. Hyperarousal is a state of persistent fight-or-flight activation across physiological, cortical, and cognitive-emotional domains [121]. Additionally, altered or disrupted activity of the resting-state functional network, particularly the default mode and salience networks, which are key components of the dynamic pain connectome, may further contribute to this association [73]. Together, the prevalence trajectory of photosensitivity and threshold findings, photosensitivity could serve as a marker of injury complexity, TBI comorbidities, and central sensitization rather than injury presence. It mainly reflects symptom burden and recovery, not severity, given its clear decline over three months.

It has been suggested that photosensitivity may result from depolarization of ipRGCs [76,79]. In mild TBI, it was proposed that axonal deformation and increased membrane permeability to calcium ions may occur in ipRGCs, shifting their resting membrane potential closer to the activation threshold. This heightened excitability could increase ipRGC sensitivity to light. Therefore, this may alter their inputs to the thalamic pain centers and the olivary pretectal nucleus, potentially contributing to sustained pupil constriction. However, the hypothesis was rejected, as no difference in pupillary response was found between the healthy and TBI groups using flickering blue and red light stimuli [76]. These findings are in agreement with another study that used similar methods and suggested that underlying damage is in the subcortical projection of ipRGC, and that neither blue nor red light stimulus type fully isolates ipRGC-driven components of the pupil response, as multiple receptors contribute to it [79].

However, these two studies contrast with another study which used a binocular pupillometer under white, red, and blue light conditions in mild TBI. The authors attributed the reduction in 6SPSD under blue light to a weaker ipRGCs-driven response, proposing it as a potential objective biomarker for photosensitivity in mild TBI. Within the mild TBI group, larger baseline pupil diameter and faster re-dilation under white/red conditions were also found in photosensitive individuals compared with non-photosensitive individuals, and the authors attributed this to damage to parasympathetic control [78]. In pediatric settings, a retrospective study did not replicate the findings from adults above [80]. In this study, photosensitivity had no impact on the measured pupillary metrics, suggesting that the correlation observed in adults does not apply in children. Taken together, these inconsistent findings indicate that further studies are required to assert the role of pupillometry in assessing photosensitivity after TBI.

Beyond photosensitivity, pupillary abnormalities could triage those with moderate and severe TBI. An NPi-200 score below 3 could help identify those at risk of neuro-worsening, with high specificity (92%) [81], and identify those who required emergent intervention in two reports [92]. This is consistent with another report showing that NPi scores below 3 on admission could predict unfavorable neurological outcomes in a cohort of individuals with different severities of TBI [91]. However, these metrics also had low sensitivity (51%), suggesting that normal NPi scores cannot rule out neuro-worsening. The authors attributed the low sensitivity to variations in age, TBI severity, time of arrival at the emergency department, and interventions [81].

A related study linked reduced NPi score to poor functional outcomes, increased intracranial pressure, and a higher likelihood of emergency procedures, while reduced dilation velocity was independently associated with loss of consciousness and need for emergency intervention. Faster constriction velocity was linked to better three-month functional outcomes, suggesting that it can predict good recovery. This was limited to the fact that the majority of participants had moderate and severe TBI, suggesting limited generalizability to mild TBI and typical concussion [84]. Taken together, findings from these studies suggest that NPi-200 can help triage individuals with moderate and severe TBI, reflect severity, and may predict short-term prognosis. However, it should not be used as a standalone test given its low sensitivity.

It has also been shown that pupillary metrics changed in asymptomatic high school football athletes with sub-concussion, even when the HHI was below the threshold, suggesting that NPi-200 scores could reflect subtle brain damage that conventional methods cannot detect without advanced imaging, based on findings [93]. However, this evidence comes from a specific population, and whether it applies to adults or other types of sports injuries remains unclear.

Using a PLR-3000 pupillometer, three studies reported inconsistent findings [85,94,95]. The female hockey athlete study, with a small sample size, found no differences [85], contrasting findings from a study with a larger sample size in individuals with mild TBI [94]. The study found a reduction in constriction velocity in those who present early, in addition to a link between symptom severity and post-illumination anisocoria. This suggests that dynamic pupil response may reveal subtle asymmetric dysfunction after concussion in those with more recent active injury [94]. In a separate study, pupillary metrics may help predict when cadets with mild TBI could return to duty [95]. Constriction velocity measured 48 h after injury could forecast return to activity or duty within 21 days, indicating its potential as a predictive marker, while dilation velocity might signal readiness for full, unrestricted activity. However, this comes from a single study, which requires replication. Overall, the usefulness of pupillometry in mild TBI varies across studies; it is most valuable when measured early and linked to outcomes like symptom severity or time to return to duty, rather than as a broad screening tool. More large-scale, standardized research focused on the early period is needed to identify which measures are truly helpful. Importantly, all three studies examined mild TBI, unlike the NPi-200 studies, which were focused on more severe brain injuries. This raises questions about whether these device findings can be applied across different TBI severity levels.

Pupillary metrics generated by NPi-200 and PLR-3000 have also been utilized in the pediatric population with concussion and yielded conflicting results, particularly in acute settings [80,96,97]. Using PLR-3000, a small minimum pupil diameter was only found in the left eye, along with worsening symptoms and cognitive and balance scores, which improved by three months, in adolescents aged between 12 and 18 years. The authors did not justify the finding of the left eye. However, this could be due to a small sample size or statistical error [96]. Using a similar device, another study on athletes with concussion found larger pupil diameters, greater percentage constriction, and faster constriction/dilation velocities, suggesting an overreactive sympathetic response and autonomic dysfunction [97]. This contrasts with a study on adults with mild TBI [94], which may be attributed to neurodevelopmental differences and injury-related variation [122]. Female adolescents showed prolonged pupillary redilation compared to males, suggesting a possible sex-based difference in autonomic recovery [97].

In modern times, smartphone applications have also been proposed to assess pupillary abnormalities and have shown promising diagnostic potential. Using PupilScreen, members of the neuro-intensive care unit were able to identify those with severe TBI with high accuracy; however, its utility remains undetermined in mild and moderate TBI [87]. In a separate study, PupilScreen demonstrated higher diagnostic accuracy than the NPi-200 score. Although again it is only in severe TBI, and this finding requires replication [86]. Applying machine learning to PupilScreen-derived data proved effective for identifying individuals with sport-related concussion using latency, diameter, and constriction velocity. This study is limited by a small, male sample size and the inability to measure the mean dilation velocity after the light stimulus is ended [98].

Another application, the Reflex-Pro PLR analyzer, for iPhone users, is also able to distinguish those with sport-related concussion even after one year of the injury, along with identifying those with photosensitivity, suggesting that smartphone-based pupillometry may retain diagnostic value even in the chronic phase, not just acutely.

Comparing smartphone-based applications with the pupillometer revealed that the NPi-200 pupillometer is the most extensively studied device, with evidence that it can detect moderate and severe TBI but with low sensitivity, suggesting that normal results cannot rule out neuro-worsening. Its potential role in sub-concussive and mild TBI is less established, with only preliminary evidence from a single cohort of high school football athletes [93].

The PLR-3000 offers additional pupillary metrics beyond NPi alone and has been applied in sports, pediatric concussion settings, and military cadet settings, but findings across studies have been inconsistent, and its utility appears age-dependent, which requires further investigation. Smartphone-based applications represent the most accessible and scalable option, requiring no dedicated hardware. Early studies on PupilScreen reported accuracy comparable to or exceeding that of the NPI-200 for distinguishing individuals with severe TBI. However, this evidence remains preliminary, drawn from a single study and not examined across different TBI severities. Furthermore, these apps lack the standardized, validated scoring framework of NPi-200 pupillometers.

Overall, device selection likely depends on clinical needs. The NPi-200 pupillometer is best suited for structured emergency triage of moderate-to-severe TBI, while smartphone applications such as the Reflex-Pro analyzer may be suitable for field-based or sports-setting screening where portability is prioritized, pending further validation. The PLR-3000’s inconsistent findings across populations suggest its utility may be setting- or age-dependent, warranting further investigation before broader adoption.

Beyond pupillometry, retinal imaging has been studied in TBI. Changes in retinal structures occur following TBI with a consistent pattern, as found across the reviewed studies [26,99,106,107]. Initial acute thickening is followed by progressive thinning, with considerable variability in timing and magnitude, when patients with mild TBI show measurable changes [99]. This matches a study with follow-ups showing that RNFL thinning increases from three to six months post-injury and then stabilizes at a similar reduced level through one year. Visual field loss peaked after three months and remained stable for six months and one year, and is associated with RNFL thinning, supporting the relationship between retinal damage and visual field impairment in mild TBI [26]. Similarly, in sport settings, initial macular thickening followed by RNFL thinning was found in boxers who were exposed to repetitive sub-concussive head trauma [106,107]. This was attributed to neuroinflammation, subtle edema, followed by retinal degeneration [106].

However, these studies conflict with a recent study that found no difference in retinal layer thickness or volume between individuals with mild or moderate TBI and healthy individuals [102]. But after excluding female participants, male participants had reduced volume and thickness of GC-IPL, along with increased volume and thickness within 3 mm and 6 mm of the OPL, suggesting that retinal assessment could be sex-dependent. These changes were correlated with the time of injury, in agreement with studies above, and attributed to the secondary wave of the biomechanical cascade. One proposed explanation of the lack of change in female participants is that mitochondria in females are more efficient in oxidative phosphorylation, have lower oxidative stress, and have greater expression of antioxidant enzymes compared to those in males. However, evidence on sex differences in TBI outcomes remains inconsistent, suggesting that these findings require further validation [123,124,125].

Single or multiple TBIs did not show a significant association with changes in retinal structures [103]. At the same time, duration since injury did, and appears to play a role in retinal degeneration. Progressive thinning of RNFL occurred between 42 and 150 days and was associated with TBI severity, as observed in those with a Marshall score of II [103]. This is in agreement with the study discussed above [26]. Emphasizing the value of long-term follow-up, a study assessed those with chronic TBI who had the injury between 3 and 35 years. The study showed structural damage of retinal layers alongside persistent mild symptoms, indicating that retinal damage remains chronic rather than fully resolvent and may reflect neurodegeneration [104].

Furthermore, retinal microvascular changes appear to follow different patterns depending on time since injury, severity, number of TBIs, and associated comorbidity [100]. In chronic (2.7 years post-injury) mild-to-moderate TBI, reductions in macular and foveal vasculature occurred without structural damage of RNFL and GC-IPL, and the magnitude is influenced by comorbid PTSD [100]. This contrasts with previous studies [26,102], which showed thinning of RNFL and GC-IPL on longitudinal follow-up. Changes in choroidal vasculature index reflect the severity and number of TBIs. A higher vascular index was observed in moderate compared to mild TBI, which may indicate changes in autonomic regulation of choroidal blood flow. Those with multiple injuries had a lower vascular index, which the authors attributed to the accumulation of Tau proteins in the vasculature [100]. In a separate longitudinal study, neither vascular nor structural changes were detectable when individuals with moderate-to-severe TBI were assessed first. However, on longitudinal follow-ups between two and six months, reduction in macular perfusion occurred and correlated with GCL thinning, suggesting that vascular and structural pathology are interlinked and may evolve over time [105].

Moreover, thickening of the Henle fiber layer observed in individuals with mild-to-moderate TBIs has been attributed to potential damage to the deep capillary plexus or to Müller cell activation in response to retinal stress. It remains unknown whether similar thickening would occur after a single TBI, as this study included only those with multiple injuries. In the same cohort, a reduction in light-adapted a-wave amplitude suggests impairment of off-cone bipolar cell responses, although there was no direct correlation with Henle layer thickening [101]. This may suggest that damage to both structures does not share an underlying mechanism.

OCT is a large machine, and it would be favorable to develop a portable device for use in sports. There was an attempt in a trial to use a portable OCT to assess retinal characteristics in unconscious and intubated patients with severe TBI; however, they failed to implement their protocol. This is a promising tool. However, it should first be tested in mild or moderate TBI [126].

Taken together from the discussed studies, retinal OCT is a valuable tool for monitoring and follow-up rather than an acute diagnostic tool. Single-timepoint assessment appears unreliable, while longitudinal follow-ups can detect meaningful structural and vascular damage and could reflect the association with neurodegeneration. Factors that should be considered include sex, time post-injury, and injury severity.

Ultimately, no single ocular biomarker category serves all the roles of diagnosis, monitoring, and recovery prediction. However, each can serve at a distinct point in TBI care. Orbital fractures are best suited as an indicator for acute diagnostic screening, especially for occult mild TBI in patients presenting with facial trauma.

DED, ocular surface assessment, and tear film biochemistry remain the least developed category across the three roles, retaining great potential to extract biomarkers, particularly from the tear film considering its proximity to the corneal nerve and corneal immune cells, and the non-invasive nature of tear collection compared to vitreous humor. Valuable biomarkers that have shown promise in ocular biofluids include CGRP, GFAP, NfL, and tTau.

NPi-200 pupillometry seems to offer the strongest evidence for acute triaging of those with moderate and severe TBI. One NPi-200 metric, constriction velocity, could predict recovery and deserves further study. PLR-3000 and smartphone-based application pupillometry have the potential to monitor and predict return to duty in field-based activities and in sport settings in those with mild TBI, although further validation is pending.

Photosensitivity assessment using different methods is the most valuable marker for recovery trajectory and injury complexity rather than a diagnostic threshold. Combining a battery of photosensitivity tests to define their repeatability and reproducibility in single and repetitive mild TBI would be valuable.

Assessment of retinal characteristics using OCT is appropriate for chronic and longitudinal follow-up rather than acute diagnosis.

## 5. Conclusions

TBI impacts ocular structures and functions, and some of these manifestations may provide non-invasive insight into underlying brain injury. However, not all ocular manifestations are true biomarkers. Orbital fractures are structural lesions that co-occur with TBI and do not reflect an ongoing biological process. However, occult fractures could help as a diagnostic indicator for mild TBI, as they might reflect changes in brain activity, although further studies are needed. Among the remaining biomarkers, no single one covers all pillars of detection, monitoring, and recovery prediction, nor does any single one capture severity and symptom burden. Instead, different markers map into different stages of the clinical course. The NPi-200 pupillometer is a valuable tool in triaging those with moderate and severe acute cases, while photosensitivity and retinal OCT better serve in monitoring and longitudinal follow-ups. PLR-3000 and smartphone applications require further research and could help in field-based activity. The ocular surface, including corneal nerves, corneal immune cells, and tear film biochemistry, remains unexplored and represents the clearest direction for future work.

## 6. Limitations

Several limitations exist in this review. First, it is a narrative review and not a systematic review, and a formal risk assessment was not conducted. Second, the reviewed studies are varied in design, sample size, TBI severity and type of population. In addition, multiple studies did not stratify TBI severity, which limits the ability to generalize the outcome. Also, some findings come from a single study and require replication, which weakens the ability to draw conclusions regarding specific markers.

## Figures and Tables

**Table 1:** Types of orbital fractures and their relationship to TBI.

Trauma	Study Design	Type of Orbital Fracture	Main Finding	Reference
Maxillofacial trauma (n = 100)	Prospective	Fractured mandible	The incidence of TBI was 67%	Joshi UM et al. [22]
Facial fractures (n = 218)	Retrospective	Zygomatic, Le Fort II, maxillary, nasal, mandibular fractures	Associated with TBI severity (*p* < 0.05, except zygomatic arch at *p* = 0.096)	Nawi, M et al. [58]
Facial fractures (n = 171)	Retrospective	Le Fort II and III fractures	Associated with neurological intervention	Lucke-Wold B et al. [60]
Ocular trauma (n = 125,000)	Retrospective	Closed orbital fracture, floor fracture, open floor fracture.	65% of them had associated TBI (mainly mild)	He C et al. [62]
		Floor fracture	Associated with severe TBI	
Maxillofacial trauma (n = 90)	Prospective	Frontal and zygomatic bone fractures	Associated with TBI (*p* < 0.001).	Navaneetham R et al. [63]
Orbital wall fracture (n = 677)	Retrospective	Supervisor wall and frontal bone fractures using the CT scan	Independent risks for concomitant intracranial injury	Lee H et al. [64]
Head injury (n = 508)	Prospective	Naso-orbito-ethmoid and zygomatic-orbital complex are the most common ones using CT scan and MRI	Orbital fractures found in 30%, 86% strongly associated with TBI	Rekha K [65]
Orbital fractures	Prospective	Orbital fractures (utilizing MRI)	Reduced density of gray and white matter	Ji L et al. [66]
Orbital fractures	Prospective	Orbital fractures (utilizing fALFF)	Lower fALFF in left anterior cingulate gyrus and right superior temporal gyrus	Kang M et al. [67]

**Table 2:** Findings of ocular surface studies in those with TBI.

Type of TBI	Study Design	Parameters or Conditions	Main Finding	Reference
Mixed severity (n = 53)	Prospective	OSDI = 20 and ocular surface staining (*p* < 0.005). No changes in TBUT and osmolarity (*p* > 0.005)	Dry eye disease should be managed in TBI, and a battery of tests should be combined.	Cockerham GC et al. [38]
TBI (n = 160)	Retrospective	Dry eyes, blepharitis, chalazion	Dry eyes occur in 15.5% and blepharitis in 18%	Rutner D et al. [70]
TBI (n = 125,000)	Retrospective	Dry eyes, ocular surface pain, chronic pain syndrome, headache, depression, PTSD	Dry eyes (37%), ocular pain (15%), and tear film dysfunction frequently occur in TBI.	Lee C et al. [23]

**Table 3:** Studies on photosensitivity and TBI.

Type of TBI (n)	Study Design	Tool Used	Main Finding	Reference
No mTBI (n = 321) mTBI (n = 320)	Cross-sectional study	NSI questionnaire	mTBI (n = 185) reported moderate to severe photosensitivity	Jotie, J.M., et al. [73]
mTBI (n = 293)	Cross-sectional study	Rivermead Post-concussion Symptoms questionnaire	mTBI (n = 124) reported photosensitivity	Shepherd, D., et al. [77]
No TBI (n = 162), mTBI (n = 186), moderate TBI (n = 34), severe TBI (n = 11)	Cross-sectional study	Photosensitivity analyser	Reflect complexity	Balba, N.M., et al. [72]
Mild TBI (n = 32) Healthy individuals (n = 40)	Cross-sectional study	Binocular pupillometer (White/red/blue light)	Reduced 6SPSD could indicate ipRGC weak response	Truong, J.Q., et al. [78]

**Table 4:** Studies on pupillary response using digital pupillometers and mobile phone applications.

Type of TBI and (n)	Study Design	Biomarker	Tool Used	Main Finding	Reference
mTBI (n = 32) Healthy individuals (n = 40)	Cross-sectional study	Pupillary parameters	Pupillary light reflex (Neuroptics DP-2000)	Increased maximum diameter, minimum diameter, maximum dilation velocity, T50, T75, decreased 6 s post-stimulus pupil diameter *	Truong J et al. [78]
TBI (n = 95)	Prospective-observational study	NPi score	NPi-200 (Neuroptics, Inc., Irvine, CA, USA)	(n = 35) experienced neuro-worsening with initial (NPi < 3). Association between neuro-worsening and NPi score ≤ 3	Trent, T., et al. [81]
TBI (n = 36)	Prospective study	Dilation velocity	NPi-200 (Neuroptics, Inc., Irvine, CA, USA)	Low dilation velocity was observed in 50% of those who later had loss of consciousness (n = 24)	Traylor, J.I., et al. [84]
TBI (n = 100)	Retrospective-observational study	NPi score	NPi-200 (Neuroptics, Inc., Irvine, CA, USA)	NPi < 3 was observed only in 30% of those who developed unfavorable outcomes.	Luz Teixeira, T., et al. [91]
Emergent interventional group (n = 27) Noninterventional group (n = 9)	Prospective pilot study	NPi score	NPi-200 (Neuroptics, Inc., Irvine, CA, USA)	NPi < 3 Lower constriction velocity and dilation velocity	El Ahmadieh, T.Y., et al. [92]
Athletes (n = 13: 8 had one sustained concussion, 5 had two sustained concussions). (Pre-season, after HHI, midseason and end of the season)	Prospective observational cohort study	NPi score	NPi-200 (Neuroptics, Inc., Irvine, CA, USA)	Reduced dilation velocity and maximum constriction velocity. Reduced percent change in pupil diameter after HHI compared to midseason.	Joseph, J.R., et al. [93]
Female athletes (n = 33: 12 had a history of concussion, 21 had no history of concussion).	Cross-sectional cohort study	PLR metrics	PLR-3000 pupillometer (Neuroptics, Irvine, CA, USA)	No differences.	Murphy, R., et al. [85]
mTBI (n = 162) Investigate the relationship between QP values and symptom severity.	Observational study	PLR metrics	PLR-3000 pupillometer (Neuroptics, Irvine, CA, USA)	Reductions in average and maximum constriction velocity in those who presented early (<2 weeks) compared to those who presented late (>2 weeks)	Bashir, M.M.I., et al. [94]
Cadets (n = 64) Baseline (T0), 48 h post-injury (T1), Symptom-free (T2), released for unrestricted duty (T3).	Prospective cohort study	PLR metrics	PLR-3000 pupillometer (Neuroptics, Irvine, CA, USA)	End-initial pupil diameter (T1), maximum constriction velocity (T1), average dilation velocity (T3)	Dengler, B.A., et al. [95]
Children (5–11 years) with concussion (n = 28) and controls (n = 29) Investigate those who are at risk of persistent symptoms. Assessed 72 h post-injury, two weeks, and after three months.	Prospective case–control study	PLR metrics, concussion symptoms (PCSI), postural stability, and neuropsychological function	PLR-3000 pupillometer (Neuroptics, Irvine, CA, USA)	No significant difference in pupillary metrics. Worse symptoms in those with a concussion.	Heyming, T., et al. [96]
Children (12–18 years) with concussion (n = 36) controls (n = 33) Investigate those who are at risk of persistent symptoms. Assessed 72 h post-injury, two weeks, and after three months.				Reduced minimum pupil diameter in those with concussion, no change in other pupillary metrics	
Sport-related concussion (n = 98, 12–18 years), healthy individuals (n = 134). Measurement taken 28 days post-injury	Prospective cohort study	PLR metrics	PLR-3000 pupillometer (Neuroptics, Irvine, CA, USA)	Greater values of maximum pupil diameter, minimum pupil diameter, percentage constriction, average constriction velocity, peak constriction velocity, average dilation velocity, peak dilation velocity, and T75. Longer T75 in females compared to males.	Master, C.L., et al. [97]
Severe TBI (n = 6, 36 curves), healthy individuals (n = 48, 84 curves).	Cross-sectional study	Two-dimensional curves represent the relationship between time and changes in pupil diameter.	PupilScreen	92% of curves from severe TBI were distinguished from those of healthy individuals	Maxin, A.J., et al. [87]
Severe TBI (n = 33) vs. healthy individuals (n = 132). To compare PLR parameters and NPi.	Cross-sectional study	PLR parameters and NPi score	PupilScreen and NPi-200 pupillometer	Max, Min, %, Lat, CV, MCV, DV NPi Higher specificity and sensitivity of PLR compared to NPi	Maxin, A.J., et al. [86]
Athletes with concussion and photosensitivity (n = 50) vs. healthy individuals (n = 50)	Cross-sectional study	Pupillary parameters	Reflex-Pro analyser-iPhone-based pupillometer	Decreased average and maximum constriction velocity, rapid dilation amplitude in those with concussion and photosensitivity	Dutta, P., et al. [90]

* The only parameter found to differentiate photosensitive individuals compared to non-photosensitive individuals with TBI.

**Table 5:** Studies on retinal structures using OCT.

Type of TBI (n)	Study Design	Biomarker	Tool Used	Main Finding	Reference
mTBI (n = 63), healthy individuals (n = 61).	Longitudinal cohort study	RNFL thickness	SD-OCT	Initial thickening at baseline, followed by a reduction in mTBI compared with healthy individuals. This could be due to retrograde degeneration in the visual cortex or post-geniculate visual radiation	Gilmore, C.S., et al. [99]
mTBI (n = 60). RNFL and visual field assessed at 7 days, 3 months, 6 months, and 1 year post-mTBI.	Prospective-observational study	RNFL thickness	SD-OCT	31% of the participants showed a reduction in RNFL at 3 months, progressed at 6 months, and no change after 1 year. Significant association between RNFL dropout and visual field loss. Thinning of RNFL can be an early biomarker for visual field defects	Kumar Das, N. and M. Das. [26]
mTBI with LOC (n = 35), mTBI without LOC (n = 25), moderate TBI (n = 6), healthy individuals (n = 37).	Cross-sectional cohort study	Thickness and volume of GC-IPL, INL, OPL, ONL, RPE.	SD-OCT	No changes. But after excluding female individuals, reduced thickness and volume of GC-IPL and increased thickness and volume of OPL in those with TBI, compared to healthy individuals.	Karthik, N., et al. [102]
mTBI (n = 48), moderate TBI (n = 63), severe TBI (n = 24). Attended two visits. Single vs. multiple TBIs.	Prospective cohort study	Thickness of RNFL and GCL	SPECTRALIS^®^ Heidelberg OCT2	No changes in RNFL between those with single or multiple TBIs. Based on the limit of agreement test–retest variability: Thinning found in one sector in GCL and all sectors of RNFL at the follow-up. Subclinical changes in the retinal layers are associated with TBI severity.	Laws, E., et al. [103]
Veterans with mTBI (n = 19) had injury > 6 months. Healthy individuals (n = 4).	Retrospective study	Thickness of pRNFL, GCL and subfoveal choroid.	SD-OCT	Thinning in the temporal pRNFL and subfoveal choroid in mTBI compared to healthy individuals. Thinning of mGCL could distinguish those with blunt head injury from those with injury caused by blast.	Chan, J.W., et al. [104]
TBI (n = 44), healthy individuals (n = 36)	Case–control study	FAZ, vascular density, perfusion density, choroidal vascularity index,	SD-OCTA with AngioPlex	Reduced circularity of FAZ, vessel density, and perfusion for 3.0 mm × 3.0 mm in those with TBI. Those with PTSD had reduced circularity of the FAZ, vessel density, and perfusion at 3.0 mm × 3.0 mm and 6.0 mm × 6.0 mm. Increased choroidal vascularity index in those with mTBI without LOC compared to moderate TBI. Lower choroidal vascularity index in those with multiple TBIs compared to those with a single one.	Justin, G.A., et al. [100]
Moderate-to-severe TBI (n = 21) were examined for less than 14 days post-injury and again after 2 to 6 months, in addition to healthy individuals (n = 18)	Prospective observational case series	Thickness of GCL, superficial vascular plexus and intermediate capillary plexus	SPECTRALIS Heidelberg OCT2	Post-injury, no difference. Severe GCL thinning at the follow-up visit after 148 days was observed in 50% of participants. Reduced perfusion was found in those with TBI compared to controls.	Hepschke, J.L., et al. [105]
mTBI (n = 25) and healthy individuals (n = 30).	Prospective case–control study	Phase retardation and thickness of Henle fiber layer (HFL).	SD-OCT and laser polarimetry	Increased thickness of HFL in TBIs. Reduced the a-wave in light and dark adaptation.	Stern-Green, E.A., et al. [101]
Boxers (n = 16). Baseline and two visits. Healthy individuals (n = 20).	Prospective longitudinal study	RNFL and the macula	SD-OCT	Within TBI, increased macula thickness and changes in the RNFL. Compared to healthy individuals, reduced parameters were found in TBI.	Childs, C., et al. [106]
Athletes (n = 46), healthy individuals (n = 104)	Cross-sectional study	RNFL and GCL complex	SD-OCT	Thinner RNFL in athletes and boxers compared to healthy individuals. Thinner average GCC in boxers compared to healthy individuals.	Leong, D., et al. [107]

**Table 6:** A summary of the consistency of ocular markers across the reviewed studies.

Marker Category	Evidence Base	Evidence Level	Consistency Across Studies
Orbital fracture co-occurrence with TBI	Retrospective studies	Low	Consistent and replicated
Occult fracture linked to changed brain activity	Two observational studies	Low	Unreplicated
Molecular biomarkers in tears and vitreous humor	Three observational studies	low	Unreplicated
DED prevalence and TBI association	Three retrospective and observational studies	Low	Inconsistent findings
Photosensitivity	Four observational studies and a meta-analysis	Low	Consistent and replicated
NPi-200 pupillometer (triage/severity)	Six studies observational studies	Low	Consistent and replicated
PLR-3000	Three observational studies	Low	Inconsistent findings
Smartphone applications	Four observational studies	Low	Unreplicated
Retinal structural changes using OCT	Observational studies	Low	General pattern replicated
Retinal OCTA vascular changes	Two observational studies	Low	Unreplicated

## Data Availability

No new data were created or analyzed in this study. Data sharing is not applicable to this article.
